# Genome sequencing-assisted identification and the first functional validation of *N*-acyl-homoserine-lactone synthases from the Sphingomonadaceae family

**DOI:** 10.7717/peerj.2332

**Published:** 2016-08-30

**Authors:** Han Ming Gan, Lucas K. Dailey, Nigel Halliday, Paul Williams, André O. Hudson, Michael A. Savka

**Affiliations:** 1 School of Science, Monash University Malaysia, Bandar Sunway, Selangor, Malaysia; 2 Genomics Facility, Tropical Medicine Biology Platform, Monash University Malaysia, Bandar Sunway, Selangor, Malaysia; 3 Thomas H. Gosnell School of School of Life Sciences, Rochester Institute of Technology, Rochester, NY, USA; 4 School of Life Sciences, Centre for Biomolecular Sciences, University of Nottingham, Nottingham, UK

**Keywords:** Acyl-homoserine lactones, LuxIR, Quorum-sensing, Two-dimensional thin-layer chromatography, *Novosphingobium*, Phylogenetic, Sphingomonadaceae, *N*-acyl-homoserine lactone synthases, Whole genome sequencing

## Abstract

**Background:**

Members of the genus *Novosphingobium* have been isolated from a variety of environmental niches. Although genomics analyses have suggested the presence of genes associated with quorum sensing signal production e.g., the *N*-acyl-homoserine lactone (AHL) synthase (*luxI*) homologs in various *Novosphingobium* species, to date, no *luxI* homologs have been experimentally validated.

**Methods:**

In this study, we report the draft genome of the *N*-(AHL)-producing bacterium *Novosphingobium subterraneum* DSM 12447 and validate the functions of predicted *luxI* homologs from the bacterium through inducible heterologous expression in *Agrobacterium tumefaciens* strain NTL4. We developed a two-dimensional thin layer chromatography bioassay and used LC-ESI MS/MS analyses to separate, detect and identify the AHL signals produced by the *N. subterraneum* DSM 12447 strain.

**Results:**

Three predicted luxI homologs were annotated to the locus tags NJ75_2841 (NovI_Nsub1_), NJ75_2498 (NovI_Nsub2_), and NJ75_4146 (NovI_Nsub3_). Inducible heterologous expression of each *luxI* homologs followed by LC-ESI MS/MS and two-dimensional reverse phase thin layer chromatography bioassays followed by bioluminescent ccd camera imaging indicate that the three LuxI homologs are able to produce a variety of medium-length AHL compounds. New insights into the LuxI phylogeny was also gleemed as inferred by Bayesian inference.

**Discussion:**

This study significantly adds to our current understanding of quorum sensing in the genus *Novosphingobium* and provide the framework for future characterization of the phylogenetically interesting LuxI homologs from members of the genus *Novosphingobium* and more generally the family Sphingomonadaceae.

## Introduction

Quorum sensing (QS) is commonly employed by bacteria to monitor population cell density to synchronize gene expression (Fuqua, Winans & Greenberg, 1994; Miller & Bassler, 2001; Schuster et al., 2013; Waters & Bassler, 2005). In one type of QS system from Gram-negative bacteria, the bacteria produce and detect chemical signals called *N*-acyl-homoserine lactones (AHLs). These signals are produced by an AHL synthase, a member of the LuxI-type protein family, and are usually detected by a transcriptional regulator belonging to the LuxR-type family. A typical AHL-QS system contains an AHL synthase (LuxI) and the transcriptional activator/repressor protein (LuxR) that are usually in a genomic context regarding proximity (Fast & Tipton, 2012). Upon reaching a concentration threshold that corresponds to a given the cell density, the AHL signal(s) is detected by the cognate LuxR and activates population-wide-responses leading to the coordination of gene activation or repression. In Gram-negative bacteria, AHL-dependent QS regulation is used to regulate diverse responses such as; the production of bioluminescence, the activation of virulence factors, conjugation, the production of antimicrobial metabolites and the production of polysaccharides (Fuqua & Greenberg, 2002; Miller & Bassler, 2001; Waters & Bassler, 2005).

Besides the presence of the canonical *luxI/luxR* pairs, many bacteria contain additional *luxR* transcriptional regulators that do not have *luxI* genes in their vicinity on the chromosome. These unpaired *luxR* genes have been coined solos or orphans and are orthologous to QS LuxR-type transcriptional regulators in that LuxR solos contain the AHL-binding domain at the N terminus and a DNA-binding helix-turn-helix (HTH) domain at the C terminus (Case, Labbate & Kjelleberg, 2008; Cude & Buchan, 2013; Fuqua, 2006; Gonzalez & Venturi, 2013; Subramoni & Venturi, 2009; Tsai & Winans, 2010). Generally speaking, the solo LuxR-type transcriptional activators increase the regulatory range by responding to endogenously produced AHLs, by listening-in or eavesdropping on exogenous signals produced by other bacteria or to alternative signals produced by the bacterial community or colonised host (Chatnaparat et al., 2012)

Members of the *Novosphingobium* genus have been isolated from a variety of terrestrial and aquatic environments, including grapevine crown gall tumor surface, *Populus deltoides* rhizosphere, marine subsurface and muddy sediments, pulp and paper wastewater, polluted waters in addition to other sites (Gan et al., 2009; White, Sutton & Ringelberg, 1996) Despite numerous successful *in-silico* identifications of sphingomonad *luxI* homolog(s) in publicly available databases, no sphingomonad *luxI* homologs have ever been experimentally validated to date (Gan et al., 2012; Gan et al., 2015; Gan et al., 2013). During our on-going work of AHL signal detection in members of the genus *Novosphingobium, N. subterraneum* DSM 12447 strain was distinguished by its ability to accumulate substantially higher amounts of AHL signals as compared to other sphingomonad strains tested in our lab.

Hence, to fill this existing gap in the quorum-sensing field, we aim to use *N. subterraneum DSM 12447* to (1) develop a two-dimensional reverse-phase thin layer chromatography (TLC) bioassay to separate and detect the multiple AHLs produced by *N. subterraneum* DSM 12477, (2) quantify and determine the structural identity of the AHL signals produced by heterologously expressed LuxI homologs using mass spectrometry analysis, (3) sequence the whole genome of strain DSM 12447 to identify its AHL biosynthetic genes and elucidate its evolutionary relationship with other LuxI homologs through Bayesian inference, and (4) functionally characterize the identified AHL synthase(s) through regulated heterologous expression in the *Agrobacterium* strain NTL4.

## Materials and Methods

### Bacterial strains, growth media and biosensor strains

*Novosphingobium subterraneum* DSM 12447 (previously *Sphingomonas subterraneum* DSM12447) was provided by Andreas Stolz (Institut fur Mikrobiologie at the Universitat Stuttgart, Stuttgart, Germany). This strain was isolated from terrestrial subsurface and was shown to catabolize a variety of natural recalcitrant and anthropogenic compounds including naphthalene, toluene, biphenyl, dibenzothiophene and fluorine (Balkwill et al., 1997; Takeuchi, Hamana & Hiraishi, 2001). The bacterial strains and plasmids used in this work are listed in Table 1.

**Table 1:** Bacterial strains plasmids and primers used in this study.

Strain	Description	Ref
*Novosphingobium subterraneum* DSM 12447	Degrades natural recalcitrant and anthropogenic compounds, AHL-producer	Balkwill et al. (1997)
*Escherichia coli* JM109	(traD36. *pro* AB +*lac* Iq, *lac*ZΔM15) end A1 *rec*A1 *hsd*R17(rk −, mk+) *mcr*A *sup*E44 *λ*- *gyr*A96 *rel*A1Δ*(lac- proAB)*	Yanisch-Perron, Vieira & Messing (1985)
*Agrobacterium tumefaciens* NTL4 (pZLR4)	pTiC58-cured derivative of C58, ΔtetRS containing pZLR4 (traR, traG::lacZ), cognate AHL: 3-oxo-C8-HSL	Luo, Clemente & Farrand (2001)
*Agrobacterium tumefaciens* A136 (pCF218)(pMV26)	Ti plasmidless host, Rf^*r*^, containing pCF218 (*traR*) and pMV26 (P*traI::luxCDABE*) cognate AHL: 3-oxo-C8-HSL	Sokol et al. (2003)
*Chromobacterium violaceum* CV026	Indicator strain for detection of alkanoyl-AHLs, derivative of wild-type strain 31532 with mini-Tn5, Km^*r*^, in the *cviI* gene cognate AHL: C6-HSL	McClean et al. (1997)
**Plasmid**	**Feature**	**Ref**
pSRKKm	Broad-host-range, Km^*R*^ IPTG-inducible	Khan et al. (2008)
pNsub1	Broad-host-range, Km^*R*^ IPTG-inducible containing the novI_Nsub1_ gene	This study
pNsub2	Broad-host-range, Km^*R*^ IPTG-inducible novI_Nsub2_ gene	This study
pNsub3	Broad-host-range, Km^*R*^ IPTG-inducible novI_Nsub3_ gene	This study
pSB401	*luxR*^+^ P*luxI-luxCDABE* Tc^*r*^ p15A ori, cognate AHL: 3-oxxo-C8-HSL	Winson et al. (1998)
pSB536	*ahyR*^+^ P*luxI-luxCDABE* Tc^*r*^ p15A ori cognate AHL: C4-HSL	Winson et al. (1998)
pSB1075	*lasR*^+^ P*luxI-luxCDABE* Tc^*r*^ p15A ori cognate AHL: 3-oxo-C12-HSL	Winson et al. (1998)
**Primer (Target)**	**Sequence and binding site^a^**	**Ref**
G9-13F (novI_Nsub1_)	JRVC01000013.1 (145,875–145,893 bp) GGAATTCCATATGCTCAACCTCACTGACG	This study
G9-13R (novI_Nsub1_)	JRVC01000013.1 (146,495–146,517 bp) CCTAGGCTAGCCATGGCTCGATTGTGATGGG	This study
G9-11F (novI_Nsub2_)	JRVC01000011.1 (118,238–118,261 bp) GGAATTCCATATGATCCATATTGTCAAAGGGTGC	This study
G9-11R (novI_Nsub2_)	JRVC01000011.1 (118,904–118,928 bp) CCTAGGCTAGCGTTATCGACAATGACATAACCGT	This study
G9-28F (novI_Nsub3_)	JRVC01000028.1 (28,716–28,735 bp) GGAATTCCATATGTTGAAAGTCACCACGCC	This study
G9-28R (NovI_Nsub3_)	JRVC01000028.1 (28,037–28,056 bp) CCTAGGCTAGCCGTAGCCATCAGTCTGCAAC	This study

**Notes.**

a Underlined nucleotide bases indicate restriction enzyme sites for cloning.

*Novosphingobium subterraneum* DSM 12447 strain was grown in tryptone soy broth (TSB), potato dextrose (PD) or R2A medium (Difco Laboratories, Detroit, MI) at 28 °C. Agrobacteria AB minimal media (Chilton et al., 1974) at 28 °C was used to grow AHL-dependent biosensor strains *Agrobacterium* NTL4 and A136. For AHL signal induction bioassays, *E. coli* JM109 and *Agrobacterium* NTL4 strains harboring empty vector pSRKKm or pSRKKm with cloned *novI* genes were grown on Luria-Bertani broth (LB) medium at 37 °C and 28 °C and supplemented with kanamycin at 25 and 50 µg/ml, respectively. For AHL signal detection bioassays, *Agrobacterium tumefaciens* NTL4 (pZLR4) and A136 (pCF218, pMV26) were grown in AB medium supplemented with 0.2% (w/v) dextrose and 0.01% (w/v) yeast extract and gentamycin (10 µg/ml) for NTL4 (pZLR4) (Cha et al., 1998) and kanamycin (25 µg/ml) and tetracycline (5 µg/ml) for A136 (pCF218, pMV26) (Sokol et al., 2003) *E. coli*-based biosensors JM109 (pSB401), JM109 (pSB1075) and JM109 (pSB536) were grown in LB media with appropriate antibiotic for plasmid maintenance (Winson et al., 1998). *Chromobacterium violaceum* CV026 biosensor was growth in tryptone yeast extract/potato dextrose (1:1) agar media for T-streak bioassays (McClean et al., 1997).

Each AHL-dependent bacterial biosensor strain used in this work along with its AHL receptor protein and cognate AHL signal is listed in Table 1. All media and growth conditions for AHL detection bioassays are as previously described by our laboratory (Gan et al., 2009; Lowe et al., 2009).

### Biosensor detection

Reverse-phase (RP) one-dimensional (1-D) TLC plates were used to determine AHL signal profiles. Concentrated acidified ethyl acetate (aEtOAc) extracts were spotted on to the C18 RP-TLC plate origin in 2-µL volumes and representing from 0.5 to 2-mL supernatant equivalents (EMD Chemicals Inc., Gibbstown, NJ). Plates were developed in a 70:30 (v/v) methanol:water mobile phase, dried and AHLs were detected as described (Scott et al., 2006). Bioluminescence produced by the *A. tumefaciens* A136 *traR*, P*traI::luxCDABE*-based biosensor strain overlaid on the chromatograms was detected with a Bio-Rad charge-coupled device (ccd) ChemiDoc MP system at two different sensitivity settings AHL signals were identified with appropriate reference compounds. This involves determining and comparing retardation factors (Rf) of unknown samples to AHL reference compounds (Shaw et al., 1997).

### Development of two-dimensional (2-D) thin layer chromatography for AHLs

The AHL extract was initially spotted onto the bottom left corner of the C18 RP-TLC plate The amount needed was estimated based on the AHL signal strength obtained from multiple independent 1-D RP-TLC runs. The spotted TLC plate was eluted with 70:30 (v/v) methanol: water as the first mobile-phase in a glass tank. The mobile-phase was allowed to rise until the top of the TLC plate before removing the plate to dry overnight. Then, the TLC plate was rotated 90° counterclockwise, placed into a tank with 25:75 (v/v) 2-propanol: water as the second mobile-phase until it reached the top of the TLC plate. After drying, the TLC plate was overlaid with TraR-dependent *Agrobacterium* biosensor strain A136 using the same procedure as used for 1-D TLCs.

### AHL identification and quantification by LC-MS/MS

#### Equipment

Chromatography was achieved using a Shimadzu series 10AD VP LC system. The column oven was maintained at 50 °C. The HPLC Column used was a Phenomenex Gemini C18 column (3.0 µm , 100 × 3.0 mm) with an appropriate guard column. Mobile phase A was 0.1% (v/v) formic acid in water, and mobile phase B 0.1% (v/v) formic acid in methanol. The flow rate throughout the chromatographic separation was 450 µL/min. The binary gradient began initially at 10% B and increased linearly to 99% B over 12 min and remained at 99% B for 1 min. A rapid decrease to 10% B occurred over 0.1 min, and stayed at this composition for 1.9 min. Total run time per sample was 15 min.

The MS system used was an Applied Biosystems Qtrap 4,000 hybrid triple-quadrupole linear ion trap mass spectrometer equipped with an electrospray ionisation (ESI) interface. Instrument control, data collection and analysis were conducted using Analyst software. Source parameters were set as: curtain gas: 20.0, ion source potential: 5,000 V, temperature: 450 °C, nebulizer gas: 20.0, and auxiliary gas: 15.0.

#### AHL standards

Synthetic standards of C4, C6, C8, C10, C12, C14, 3-oxo-C4, 3-oxo-C6, 3-oxo-C8, 3-oxo-C10, 3-oxo-C12, 3-oxo-C14, 3-OH-C4, 3-OH-C6, 3-OH-C8, 3-OH-C10, 3-OH-C12 and 3-OH-C14 AHLs were synthesised according to established procedures (Chhabra et al., 2003; Chhabra et al., 1993).

#### Sample preparation

Dried extracts were stored at −20 °C. Prior to analysis, each sample extract was reconstituted in 100 µl of methanol +0.1% (v/v) formic acid. The injection volume was 5 µl.

#### Analysis method

Initial analysis was conducted with the MS operating in precursor ion scan mode screening for precursor ions that give rise to a product ion of *m*∕*z* = 102 (a fragment ion that is common to all AHLs), upon collision induced fragmentation (Table 2). Comparison of detected peak areas with an AHL mix sample of known concentration was used to gauge a useful calibration range for the subsequent quantification of detected AHLs. Samples were rerun with the MS in MRM (multiple reaction monitoring) mode, analysing the LC eluent for specific AHLs detected in the previous analysis. The quantification was conducted by comparing peak areas of detected peaks with a six point calibration line constructed by analysing (in triplicate) mixed AHL calibration samples containing C8, 3-OH-C8 and 3-OH-C10 AHLs at 0.5, 1.0, 2.0, 5.0, 10 and 20 µM.

**Table 2:** Mass transitions used for the MRM detection of common AHLs.

Acyl chain length	Carbon 3 substitution	MRMs	Retention time/min
C4	Unsubstituted	172–102	3.18
	Oxo	186–102	2.02
	OH	188–102	1.58
C6	Unsubstituted	200–102	4.53
	Oxo	214–102	4.08
	OH	216–102	3.93
C8	Unsubstituted	228–102	5.20
	Oxo	242–102	4.66
	OH	244–102	4.49
C10	Unsubstituted	256–102	5.87
	Oxo	270–102	5.31
	OH	272–102	5.05
C12	Unsubstituted	284–102	6.65
	Oxo	298–102	5.98
	OH	300–102	5.72
C14	Unsubstituted	312–102	7.44
	Oxo	326–102	6.77
	OH	328–102	6.50

### Whole genome sequencing of *N. subterraneum* DSM 12447

Genomic DNA of strain DSM12447 was extracted using the GenElute™ (Sigma-Aldrich, St. Louis, MO, USA) and converted into next generation sequencing library using Nextera XT (Illumina, San Diego, CA, USA) according to the manufacturer’s instructions. Whole genome sequencing was performed using the MiSeq (Illumina, San Diego, CA, USA) at the Monash University Malaysia Genomics Facility. The raw data for each bacterium were error-corrected and assembled using Spades v2.5 (default setting) (Bankevich et al., 2012). The generated contigs were scaffolded and gap-closed using SSPACE and GAPFiller, respectively (Boetzer et al., 2011; Boetzer & Pirovano, 2012). Genome annotation was performed using Prokka and InterProScan5 (Jones et al., 2014; Seemann, 2014).

### Identification and phylogenetic analyses of LuxI homologs

The whole genome of strain DSM 12447 was submitted to Anti-SMASH server (Weber et al., 2015) for the identification of biosynthetic gene cluster(s) (including AHL synthase cluster). *In-silico* validation of the identified LuxI homologs was performed through protein alignment with bona fide LuxI homologs and manual inspection of the alignment for conserved LuxI homologs amino acid residues. Visualization of the gene organization was performed with EasyFig (Sullivan, Petty & Beatson, 2011) using NCBI annotated sequence as input. Protein alignment was done using MAFFT-LINSI (Katoh & Standley, 2014) and the alignment was trimmed with trimal (-gappyout) to retain as much site informative as possible (Capella-Gutierrez, Silla-Martinez & Gabaldon, 2009). Subsequently, a phylogenetic tree was inferred using phylobayes (-cat –gtr –ncat 4) (Lartillot et al., 2013). A total of four independent chains were run for 10,000 generations each. The first 1,000 trees were discarded as burn-in and a consensus tree was built based on the 50% majority rule. Mesquite was used for tree visualization and editing (Maddison & Maddison, 2015).

### Amplification and cloning of 3 putative AHL synthase genes

Primers and plasmids used in this study are listed in Table 1. Amplification of the *luxI* homologs was performed using Q5 polymerase mastermix (New England Biolabs, Ipswich, MA, USA) according to the manufacturer’s instructions. Approximately 150 ng of the purified PCR amplicons were mixed with 50 ng of pSRKKm vector (Khan et al., 2008) and double digested with NheI and NdeI (New England Biolabs, Ipswich, MA, USA) for 1 h. After heat inactivation, the digested products were purified using magnetic beads (Omega Biotek, Norcross, GA, USA) and ligated with Electroligase (New England Biolabs, Ipswich, MA, USA) for 30 min. The ligated products were transformed into *A. tumefaciens* NTL4 and *Escherichia coli* JM109 using electroporation.

### Inducible expression of *luxI* homologs and detection of AHLs from solid media

Bacterial culture (*A. tumefaciens* NTL4 and *Escherichia coli* JM109 grown for 96- and 48 h, respectively) supernatants were resuspended from LB plates supplemented with antibiotic kanamycin and filter-sterilized IPTG at 0, 10, 100 and 1000 µM containing pSRKKm with and without the cloned *luxI* homologs (Table 1) were extracted with acidified ethyl acetate (aEtOAc) (1 mL of glacial acetic acid per 200 mL of ethyl acetate) for 60 min with shaking (150 r.p.m.). The extracts were then centrifuged to separate the aqueous and ethyl acetate phases. The ethyl acetate phase was recovered and dried in a Savant Speed Vac. Twenty-, thirty- or fifty-fold concentrated extracts were prepared and used in AHL detection bioassays.

### Acyl-homoserine lactone extractions for induction assays

NTL4 (pNsub1,2 or 3) strains were grown in 20 mL of LB (50 µg/ml kanamycin) supplemented with different amount of inducer isopropyl *β*-D-1-thiogalactopyranoside (IPTG) inducer to final concentration of 0,10,100, or 1,000 µM and incubated overnight in a shaking incubator at 28 °C. The next day, 20 ml of ethyl acetate was added to each of the twelve tubes and shaken at room temperature for 2 h. The tubes were then centrifuged at 5,000 rpm for 10 min to separate the liquid layers and then the top layer of ethyl acetate was aspirated off and stored. This layer was separated into several 1.5 ml micro centrifuge tubes and the ethyl acetate evaporated off using a speed-vac. The residue in the tube was then resuspended in 75 µl of fresh ethyl acetate to bring all samples to a 20×-volume equivalent extract concentration.

## Results

### *N. subterraneum* DSM 12447 produces multiple distinct AHL signals

**Figure 1:**
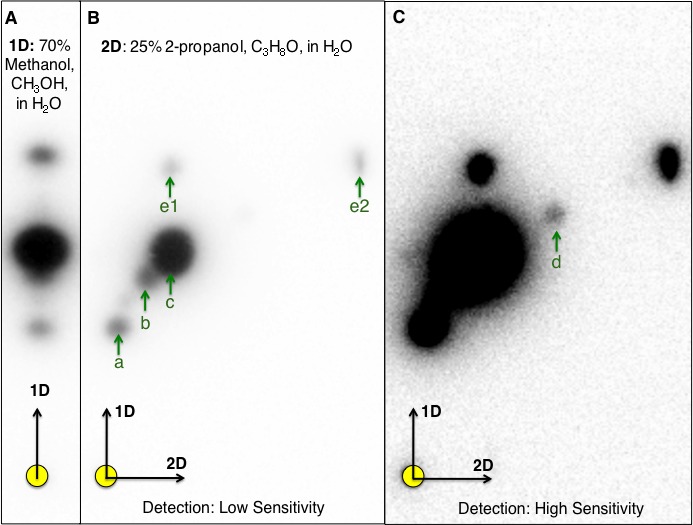
One-dimensional (1-D)- and two-dimensional (2-D)-reverse phase thin layer chromatography (RP-TLC) separation and TraR-LuxCDABE-based detection of *Novosphingobium subterraneum* AHL signals. Ten microliters of 20× extract prepared from *N. subterraneum* NBRC 16086 strain grown on solid media was spotted to the 1-D and 2-D chromatographs (circle in lower left of each image). (A) Conventional 1-D TLC showing the detection of three AHL signals by *Agrobacterium tumefaciens* A136 (pCF218, pMV28). The CCD camera setting: low at 0, Gamma at 1.0 and high at 42,500 unless noted. (B) Resolution of additional AHL signals by A. *tumefaciens* A136 (pCF218, pMV28) as a result of the development of 2-D RP-TLC separation conditions for AHLs. For (B), the CCD camera high setting was at 47,771. (C). Improved coupled charge detection (CCD) camera detection of AHL signals of the same 2-D RP-TLC overlaid as in (B), the high setting was decreased to 3,000. Arrows denote detected signal and identical alphabetical letters denote AHL signals with similar retardation factor.

Culture extracts prepared from *N. subterraneum* DSM 12447 activated three of the five AHL-dependent whole cell bacterial biosensors tested (Table 3). One-dimensional (1-D) RP-TLC separation of the culture extract followed by AHL detection using TraR-based bioluminescence biosensor led to the confident detection of three putative AHL signals (Fig. 1A). Given the lack of signal resolution presumably due to high AHL signal diversity, two-dimensional (2-D) RP-TLC was developed in this work to improve AHL separation and detection. The additional separation of AHL using 25% 2-propanol as the second mobile phase coupled with detection using a luminescence-based reporter rendered significant improvement in AHL signal detection and identification. Based on 2-D RP-TLC of extracts prepared from *N. subterraneum* DSM 12447 strain followed by AHL detection using *A. tumefaciens* A136 overlay, six distinct putative AHL signals were identified (Figs. 1B–1C).

**Table 3:** Detection of *N*-acyl-homoserine lactones by five different AHL-dependent biosensor strains.

	AHL receptor^a^				
	AhyR^b^	LuxR^b^	TraR^b^	LasR^b^	CviR^c^
*Novosphingobium subterraneum* DSM 12447	−	+ +	+ + +	−	+

**Notes.**

a AhyR, AHL receptor from *Aeromonas hydrophilia*; LuxR, *Vibrio fisheri*; TraR, *Agrobacterium tumefaciens*; LasR, *Pseudomonas aeruginosa*; CviR, *Chromobacterium violaceum*.

b Scores for bioluminescence-based biosensor detection of AHL in strain extracts: −, <2-fold higher than background levels of relative light units (RLU) bioluminescence; + > 2-fold higher than background RLUs; + + > 50 to 75-fold higher than background RLUs; + + + > 75-fold higher than background RLUs.

c Violacein pigment (purple) production in T-streak bioassays on PDA/TYE (1:1) agar media: +, visible pigment production; −, no pigment production.

### Whole genome sequencing of *N. subterraneum* DSM 12447 identified 3 putative *luxI* homologs (*novI*) that share a common *novRnovIphyH* gene synteny

**Figure 2:**
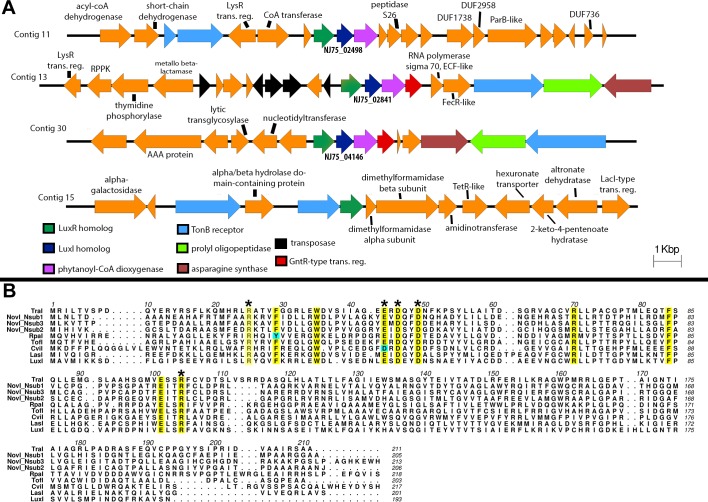
Identification and gene organization of three functional LuxI homologs and one newly identified luxR solo. (A) Alignment of three identified *Novosphingobium subterraneum* LuxI homologs with other previously reported functional LuxI homologs. Conserved sites are highlighted in yellow and cyan-colored residues indicate deviation from consensus sequence. (B) Gene organization of the identified *luxI* homologs and also a newly identified *luxR* solo.

The draft genome of strain DSM 12447 has a GC content of 63.2% and consists of 54 contigs with a total genome length of 4,885,942 bp (N_50_ of 181,386 bp). Anti-SMASH analysis (Weber et al., 2015) revealed three LuxI-type AHL synthase genes that are separately located in three different contigs (Fig. 2A). Protein alignment of the putative LuxI homologs with known LuxI homologs shows that these homologs contain the highly conserved amino acid signatures which are crucial for the function of AHL synthesis (Asterisk signs in Fig. 2B). We propose the names, NovI_Nsub1_, NovI_Nsub2_ and NovI_Nsub3_ for locus tags NJ75_2841, NJ75_2498 and NJ75_4146, respectively. Among autoinducer proteins within the genus *Novosphingobium*, NovI_Nsub1_, NovI_Nsub2_ and NovI_Nsub3_ show 62.4%, 51.1% and 62.2% protein identity to LuxI homologs of N. sp AP12 (PMI02_00996), N. sp. Leaf2 (ASE49_1606) and N. sp. AAP1 (IP65_14795), respectively. Beyong the genus *Novosphingobium*, NovI_Nsub1_, NovI_Nsub2_ and NovI_Nsub3_ show 61.5%, 100% and 60.8% protein identity to the LuxI homologs of Sphingobium sp AP49 (PMI04_04262), Sphingopyxis sp. H050 (ATE67_10720, and Sphingobium japonicum UT26S (SJA_C1-29990)), Analysis of the gene neighbourhood of all three *novI* genes reveals a conserved *novR-novI-phyH* arrangement (Fig. 2A). The gene organization of novI_Nsub1_ and novI_Nsub3_ differ slightly in that novI_Nsub3_ contains an additional convergently oriented gene coding for GntR-like transcriptional regulator directly downstream of *phyH* along with the same other genes of similar composition as in novI_Nsub1_ but in the opposite orientation. It is also worth noting that several transposase-coding genes were tightly clustered upstream of novI_Nsub3_ suggesting that novI_Nsub3_ maybe a result of replicative transposition (Fig. 2B). In addition, a *luxR* solo was also identified in contig15 based on the presence of several signature domains associated with the canonical LuxR protein IPR005143 (Autoinducer binding), IPR016032 (Signal transduction response regulator, C-terminal effector), IPR011991 (Winged helix-turn-helix DNA-binding domain), and IPR000792 (Transcription regulator LuxR, C-terminal) in the translated protein (Fig. 2B).

### Phylogenetic analysis of all functionally validated acyl-homoserine lactone synthases reveals new insight into their evolutionary relatedness

**Figure 3:**
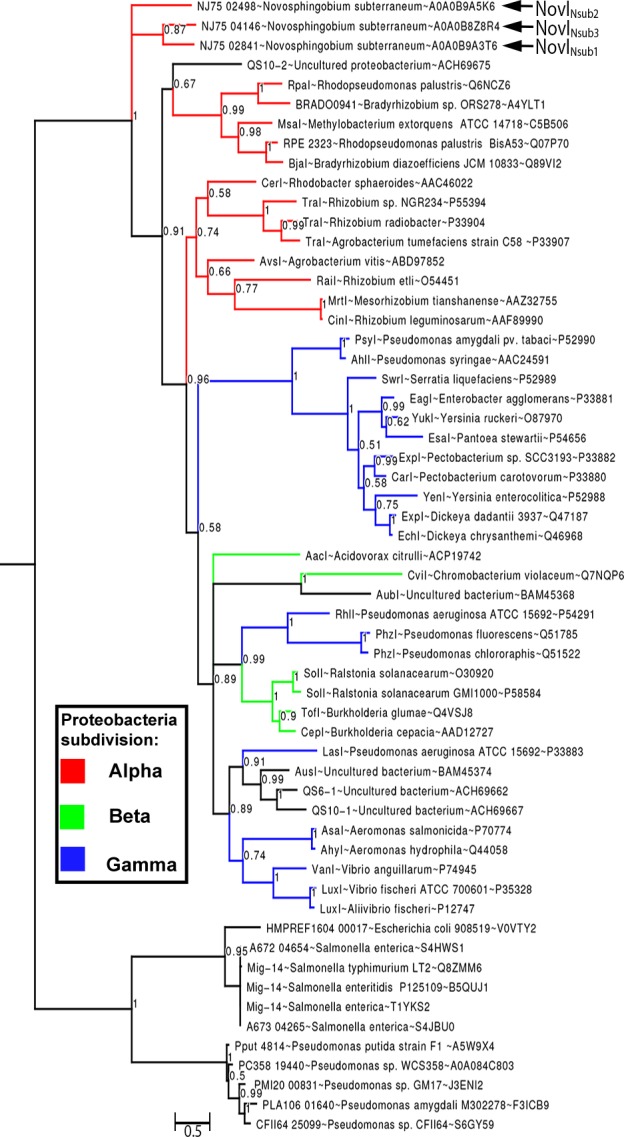
Bayesian inference of LuxI phylogeny. Support values at nodes indicate Bayesian posterior probability. Red, blue and green colored branches denote LuxI homologs from Alpha-proteobacteria, Beta-proteobacteria and Gamma-proteobacteria, respectively. The tree was rooted with Mig-14 proteins from *Salmonella* and *Pseudomonas* as the outgroup.

By rooting MAG-14 homologs as the outgroup, Bayesian inference of the newly identified NovI proteins do not exhibit close evolutionary relatedness to any of the selected LuxI homologs and instead occupy a very basal position in the phylogenetic tree. NovI_Nsub1_ andNovI_Nsub3_ formed a monophyletic clade among themselves with moderate strong posterior probability support (pp = 0.87) (Fig. 3). The relatedness of NovI_Nsub1_ and NovI_Nsub3_ as observed in the phylogenetic tree is further supported by the conservation in their gene organization (Figs. 3 and 2A). A majority of the functionally validated LuxI homologs isolated from metagenomic libraries did not demonstrate novel phylogenetic position and instead formed monophyletic clustering with known LuxI homologs with strong posterior probability support. CviI from *Chromobacterium violaceum* shared the most common ancestor with metagenome-derived AubI with maximal posterior probability support while LasI from *Pseudomonas aeruginosa* is sister taxa to the clades containing AusI, QS6-1 and QS10-1 (pp = 0.91). One notable exception is QS10-S (accession number: ACH69675) that formed a weakly supported (pp = 0.67) monophyletic cluster with the clade containing LuxI homologs from the genera *Rhodopseudomonas, Bradyrhizobium* and *Methylobacterium* which also include BjaI, an unusual isovaleryl- HSL synthase.

### First functional validation of three LuxI homologs from the family sphingomonadaceae

**Figure 4:**
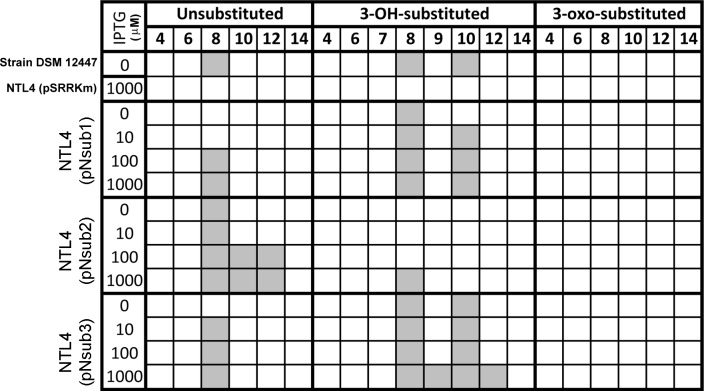
Screening of AHL signals production by individual heterologously expressed NovI homolog against a wide range of authentic standards. Colored columns indicate presence of detectable signal (>0 signal peak area unit). Numerical values in the second row indicate the acyl-chain length of AHL.

All three identified NovI homologs led to the accumulation of AHL signals in culture medium when they were heterologously expressed in *Agrobacterium tumefaciens* strain NTL4 (Fig. 4). Using an inducible expression, the effect of low-, medium- and high-expression on AHL accumulation pattern i.e., detection of additional AHL signals previously not observed in the wild type, can be better studied. In this system, the addition of inducer, IPTG, to the culture medium results in the de-repression of the cloned genes within the cells of the population (Khan et al., 2008). Out of the 20 screened AHL signals, a total of 7 AHL unsubstituted and OH-substituted signals can be identified at the highest IPTG induction of three cloned *novI*. The signals detected include C8, C8-OH and C10-OH for NovI_nsub1_; C8, C10, C12 for NovI_nsub2_ ; and C8, C8-OH, C9-OH, C10-OH and C12-OH for Nov_nsub3_. It is worth noting that, in the absence of IPTG inducer, basal levels of AHL signals were detected in the growth medium, suggesting suboptimal gene repression by the lacR repressor in *Agrobacterium tumefaciens* host. In comparison to the 2-D RP-TLC analysis (Fig. 1), LC-MS/MS analysis corroborated the presence of six AHLs and extended it to seven AHLs through inducible heterologous expression of the three individual NovI homologs of *N. subterraneum* DSM 12447 (Fig. 4).

### Dissimilar amount and ratio of major AHLs accumulation in liquid media during the heterologous expression of NovI_Nsub1_ and NovI_Nsub3_

**Figure 5:**
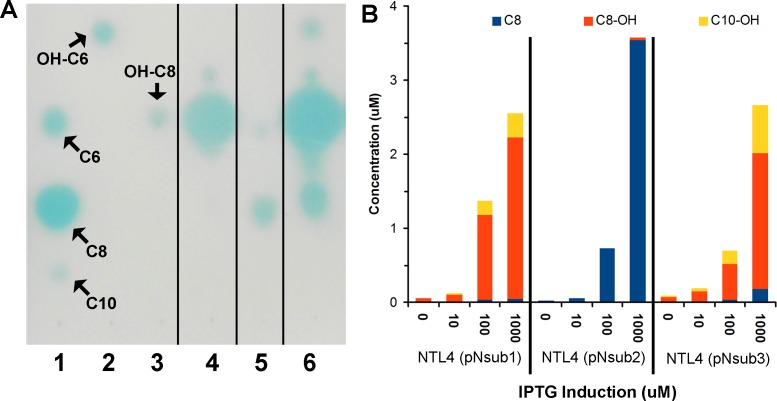
Identification and quantification of AHL signals produced through the individual heterologous expression of *Novosphingobium subterraneum* LuxI homologs. (A) One-dimensional TLC-based detection. Lane 1, Unsubstituted medium length AHL signals; Lane 2, 3-hydroxy-C6 AHL; Lane 3, 3-hydroxy-C8 AHL; Lane 4, NTL4 (pNsub1) 2 µl of 20 × EtOAc extract; Lane 5, NTL4(pNsub2) 6 µl of 20 × EtOAc extract; Lane 6, NTL4(pNsub3) 1 µl of 20 × EtOAc extract. (B) Concentration of three major AHL molecules, C8-OH, C8 and C10-OH, were calculated based on standard curves calibrated with different concentrations of authentic AHL standards.

To quantify the major AHLs (C8, C8-OH and C10-OH) produced by the heterologously expressed NovI proteins, culture extract samples from the three *novI* homologs cloned in pSRKKm and harboured in *A. tumefaciens* NTL4 were analyzed by LC-MS/MS alongside prepared samples for a six-point calibration curves ranging from 0.5 µM–20 µM for C8, C8-OH, and C10-OH. AHL signals. C8, C8-OH, and C10-OH, are the signals present in the highest concentrations in culture extracts. This approach identified and quantified the three main signals produced by the three NovI homologs showing that NovI_Nsub2_ mainly produces C8 while C8-OH and C10-OH are the major AHLs synthesized by NovI_Nsub1_ and NovI_Nsub3_. The ratio of C8-OH to C10-OH consistently differ by at least 2-fold in NovI_Nsub1_ and NovI_Nsub3_ (6.6 vs 2.8) at various IPTG induction concentrations (Fig. 5B). The strong overlap of AHL accumulation profile between NovI_Nsub1_ and NovI_Nsub3_ provides additional evidence supporting their close evolutionary relatedness.

## Discussion

In this study, we first developed a 2-D RP-TLC method to separate multiple AHLs produced by the distinct AHL-producing sphingomonad strain, *N. subterraneum* DSM 12447. Then we report for the first time, the functional validation of three *luxI* homologs from the family Sphingomonadaceae through cloning and regulated heterologous expression. The characterization of extracts of the host growth media after induction using different AHL-dependent biosensors and also by LC-MS/MS confirmed the authenticity of the synthesized AHL signals. In addition, this work is the first to demonstrate the utility of 2-D RP-TLC coupled to bioluminescence detection for the separation and more sensitive detection of multiple (and complex) AHL signals; this is especially pertinent to research laboratories that do not readily have access to LC-MS/MS equipment suited for AHL identification. In a future *in vivo* study, it would be interesting to explore if the change in AHL profiles are correlated to the alterations of a variety of growth media.

The conserved *novI-novR-phyH* synteny adds to the growing association of such a synteny with the *luxI* homologs of Sphingomonadaceae family as reported previously (Gan et al., 2013). This arrangement has also been discovered in metagenomic sampling as described by Hao and colleagues (Hao et al., 2010). In eukaryotes, PhyH is localized in the peroxisome and catalyzes the alpha-oxidation of phytanic acid to pristanic acid through the elimination of one carbon (Van den Brink & Wanders, 2006). The frequent association of *phyH* with various *novI/R* leads Hao and coworkers (Hao et al., 2010) to speculate that its transcription maybe regulated by quorum sensing. The increasing observation of *phyH* linkage with the *novI/R* warrants future work investigating its transcriptional regulation by quorum sensing and more importantly the yet-to-be described enzymatic reaction that PhyH catalyzes in bacteria.

Similarly utilizing Mig14 family protein (PF07395) from the acetyltransferase-like clan (CL0257) as the outgroup, our phylogenetic inference does not support the the basal position of clade containing the YenI, EagI, EsaI proteins (Christensen et al., 2014). Such striking differences may stem from the lack of LuxI homologs sampling from the Sphingomonadaceae family, a potential source of new phylogenetic signal, and possibly the usage of different phylogenetic inference method e.g., distance-based vs model-based. The similar neighborhood joining methods employed by Christensen et al. (2014) was also implemented in two major LuxI phylogeny studies (Gan et al., 2013; Gray & Garey, 2001; Lerat & Moran, 2004). The newly constructed Bayesian tree incorporating complex model for across-site heterogeneities in addition to improved taxon sampling represents a significant improvement over previously reported phylogenetic (Lartillot, Brinkmann & Philippe, 2007; Lartillot & Philippe, 2004; Zwickl & Hillis, 2002). That being said, the basal position of LuxI homologs from the genus *Novosphingobium* was similarly observed in a previously reported neighborhood-joining tree for LuxI homologs (Gan et al., 2013). It will be interesting to see if the tree topology will remain consistent as more LuxI homologs are being functionally validated and included into the phylogenetic analysis in the future.

The close evolutionary relationship of NovI_Nsub1_ and NovI_Nsub3_ corroborates with their overlapping AHL profile i.e., when heterologously expressed, both produce mainly C8-OH and C10-OH but at a different ratio. The presence of various genes coding for transposases upstream of novI_Nsub1_ along with its high relatedness to novI_Sub3_ suggests recent replicative transposition. The slight dissimilarity in AHL production efficiency between NovI_Sub1_ and NovI_Sub3_could be explained by an on-going “neofunctionalization” process given that the constraints of purifying selection are expected to be relaxed on duplicate gene thus allowing new evolution innovation. The transposition of *luxI* and/or *luxR* has been previously suggested in various sphingomonad strains (Gan et al., 2013) By demonstrating the functional overlap of the two *luxI* homologs, this work provides important evidence supporting the diversification of *luxIluxR* through duplication as previously hypothesized (Lerat & Moran, 2004).

## Conclusions

*Novosphingobium subterraneum* DSM 12447 accumulated different medium-length AHL compounds with a majority of them being C8-OH. Whole genome sequencing and annotation identified three *luxI* homologs in strain DSM 12447 with two of them exhibiting higher relatedness as evidenced by their monophyletic clustering, shared gene synteny and overlapping AHL signal profile based on heterologous expression in *Agrobacterium tumefaciens* NTL4. This work provides the first functional validation of LuxI homologs in the family Sphingomonadacea.

## Additional Information and Declarations

### DNA Deposition

The following information was supplied regarding the deposition of DNA sequences: This Whole Genome Shotgun project for *N. subterraneum* DSM 12447 has been deposited at DDBJ/EMBL/GenBank under the accession JRVC00000000. The version described in this paper is version JRVC01000000.
